# PD-L1 testing by immunohistochemistry in immuno-oncology

**DOI:** 10.17305/bjbms.2022.7953

**Published:** 2023-01-06

**Authors:** Semir Vranic, Zoran Gatalica

**Affiliations:** 1 College of Medicine, QU Health, Qatar University, Doha, Qatar; 2 Department of Pathology, University of Oklahoma College of Medicine, Oklahoma City, OK, United States

**Keywords:** Cancer, immunotherapy, immune checkpoint inhibitors (ICIs), predictive biomarkers, programmed cell death ligand 1 (PD-L1), immunohistochemistry (IHC)

## Abstract

Immunotherapy, based on immune checkpoint inhibitors (ICIs) targeting the programmed cell death ligand 1 (PD-L1) and/or programmed death receptor 1 (PD-1), has substantially improved the outcomes of patients with various cancers. However, only ~30% of patients benefit from ICIs. Tumor PD-L1 expression, assessed by immunohistochemistry (IHC), is the most widely validated and used predictive biomarker to guide the selection of patients for ICIs. PD-L1 assessment may be challenging due to the necessity of different companion diagnostic assays for required specific ICIs and a relatively high level of inter-assay variability in terms of performance and cutoff levels. In this review, we discuss the role of PD-L1 IHC as a predictive test in immunotherapy (immuno-oncology), highlight the complexity of the PD-L1 testing landscape, discuss various preanalytical, analytical, and clinical issues that are associated with PD-L1 assays, and provide some insights into optimization of PD-L1 as a predictive biomarker in immuno-oncology.

## Introduction

Immunotherapy, based on the use of immune checkpoint inhibitors (ICIs), has recently revolutionized the treatment and outcome of several cancer types. ICIs therapy targeting programmed death receptor 1 (PD-1), programmed cell death ligand 1 (PD-L1), and CTLA-4 have become the standard of care for several common malignancies. Anti-PD-1/PD-L1 treatment modalities include two monoclonal antibodies against PD-1 receptor (nivolumab and pembrolizumab) and three against PD-L1 (atezolizumab, durvalumab, and avelumab), all of which were approved by Food and Drug Administration (FDA) [1]. These drugs have been approved with different indications as either monotherapy or combinatorial therapy with other modalities, such as radiation therapy, chemotherapy, or other ICIs. In addition, two agents are available targeting another checkpoint regulator CTLA-4: Tremelimumab and ipilimumab [2, 3].

Despite the remarkable efficacy of the ICIs, many patients (~70%) do not respond well or develop resistance to these drugs. The response rate to ICIs varies between 15% and 30% in most solid tumors and 45%–60% in microsatellite instability high (MSI-H) cancers and malignant melanoma [4]. The use of ICIs is associated with potentially significant toxicity and side effects. Thus, in non-small cell lung carcinoma (NSCLC) treated with ICIs, the most common adverse effects are those related to endocrine, gastrointestinal, and dermatologic sites; in malignant melanoma, the most common adverse effect sites are dermatologic, hepatic, and endocrine [4, 5]. Long-term adverse effects in cancer survivors on immune system, cardiovascular functions (heart, atherosclerosis, and hypertension), neuroinflammation, and obesity have not been fully characterized but are actively investigated [6].

Therefore, development of reliable predictive biomarkers for ICIs would represent an essential selection tool. This review summarizes the status of approved and emerging predictive biomarkers for ICIs, focusing on PD-L1 expression and its quantification using immunohistochemistry (IHC). We highlight a complex PD-L1 testing landscape, highlighting preanalytical, analytical, and clinical issues that are associated with PD-L1 assays. We briefly cover regulatory issues and provide some insights into optimization of PD-L1 as a predictive biomarker in immuno-oncology.

## The role of the PD-1/PD-L1 axis in cancer surveillance and suppression

Programmed death ligand 1 (PD-L1, CD274), one of two ligands for the PD-1 receptor (the other is PD-L2, CD273), interacts with the PD-1 receptor on naïve T-lymphocytes inhibiting T-cell activation [7]. PD-L1 expression in a tumor is a sign of an inhibition of the anti-tumoral activity of the immune system and a predictor of a favorable response to the therapeutic monoclonal antibodies designed to break this inhibition and elicit antitumoral activity [8].

PD-L1 is a transmembrane receptor that interacts with PD-1 and B7.1, causing immune system suppression. PD-1 is overexpressed on T-lymphocytes following their activation and sustained during chronic stimulations, such as in chronic infections/inflammation or cancer [9]. The PD-1/PD-L1 interactions block T-lymphocyte activation, cytokine production, and cytolytic activity, causing functional downregulation or exhaustion of T-lymphocytes [9]. B7.1 receptor is overexpressed on antigen-presenting cells (APCs) and activated T-lymphocytes. PD-L1 binding to B7.1 on T-lymphocytes and/or APCs inhibits the immune responses, including inhibition of T-lymphocyte activation and cytokine production [10].

Within the tumor, expression of PD-L1 may be observed in infiltrating immune and neoplastic cells [11, 12]. The previous studies revealed that PD-L1 expression on tumor cells (TC) is associated with the downregulation of the immune system, followed by immune evasion [9]. Therefore, interruption of the PD-L1/PD-1 inhibitory axis represents an attractive therapeutic target to reactivate the T-cell response that is suppressed by the upregulation of PD-L1 in the tumor.

Currently, PD-L1 immunohistochemical (IHC) assays have the most FDA approvals as a companion diagnostic (CDx) for immunotherapy with immune checkpoint inhibitors in specific tumor types [1]; other immunotherapy predictive biomarker exist and will be briefly discussed in the next paragraph.

## Predictive biomarkers of response to immune checkpoint inhibitors

Numerous biomarkers are evaluated for the prediction of response to ICIs and three are currently approved. These are PD-L1 expression, tumor mutational burden (TMB) and DNA mismatch repair deficiency [(dMMR) and microsatellite instability-high (MSI-H)]. PD-L1 protein expression is tested by IHC. Tumor DNA-mismatch repair (MMR) protein deficiency is tested by IHC and tumor DNA microsatellite instability is tested using either PCR- or NGS-based assays. TMB is assessed using a large panel next-generation sequencing assay (NGS, currently only FoundationOne CDxTM assay) has been approved by FDA for this purpose [13].

Presence of tumor-infiltrating lymphocytes (TIL) was traditionally associated with MSI-H colorectal cancers (CRC) [14] which are frequently histopathologically analyzed in various tumors. However, it has not been formally approved as ICI therapy biomarker. In some cancers, such as breast cancer, there have been substantial efforts to standardize the assessment of TIL as proposed by the International TIL Working Group [15]. A routine assessment of TIL has also been incorporated in the fifth edition of the WHO Breast Tumors Classification [16]. Similar efforts to standardize TIL assessment have also been made in other solid tumors [17].

Other predictive biomarkers are also being intensely explored, such as PD-1, IFN-y pathway genes, IL-8, CD39+/CD8+ TIL, T-cell repertoire clonality, etc. (reviewed in [18]). None of these novel biomarkers has been approved as a predictive biomarker in immuno-oncology.

### Tumor mutational burden (TMB)

All cancers accumulate mutations (albeit at different rates), resulting in a production of novel peptides/proteins (also called neoantigens) that may be presented by major histocompatibility complex I (MHC-I) on the cell membrane of neoplastic cells. These neoantigens may be recognized by the immune cells (IC) (T-cells) as non-self (“immunogenic antigens”), triggering and provoking an immune reaction [19–21]. Notably, only a minority of these neoantigens (2–5 out of several hundred) become immunogenic, resulting in a T-cell response [19]. Consequently, the greater the TMB, the higher the likelihood of producing potentially immunogenic neoantigens and immune reactions.

TMB is defined as the number of mutations in the cancer cells. The TMB is measured by some high-throughput (NGS-based) assays, such as whole-genome or whole-exome sequencing (WGS or WES) and is reported as the number of mutations per megabase (Mutations/Mb) [22]. Both assays explore a wide range of mutations within cancer cells. Multiple WGS/WES platforms are currently available for the TMB assessment employing both non-synonymous and synonymous exonic mutations in the TMB estimation [23, 24]. Currently, only the FoundationOne CDxTM test (Foundation Medicine, Cambridge, MA, USA) is the FDA-approved assay, which includes TMB as part of its comprehensive genomic profiling panel. The Memorial Sloan Kettering Cancer Center MSK-IMPACT (Integrated Mutation Profiling of Actionable Cancer Targets, the panel of 468 genes) also received FDA authorization in 2017, but TMB as a predictive biomarker for ICIs is not included in the approval [25].

Previous studies have provided solid evidence that the tumors with a TMB ≥10 mut/Mb (TMB-high) are more likely to have a favorable response to immune checkpoint inhibitors (particularly pembrolizumab) [22]. This has been confirmed in several common cancers, such as NSCLC, urothelial carcinoma, malignant melanoma, and small cell lung carcinoma (SCLC) [19]. In two recent comprehensive pan-cancer studies exploring up to 27 different cancer types, high TMB correlated well with the response to immune checkpoint inhibitors [26, 27]. It is noteworthy that TMB depends on cancer pathogenesis and may substantially vary between and within the same/similar histologic cancer types. A good example is a Merkel cell carcinoma (MCC), a highly aggressive neuroendocrine cutaneous neoplasm. The etiology of MCC is strongly associated with two important risk factors—UV exposure and Merkel cell polyoma virus (MCPyV) positivity. Each of these risk factors causes a distinct MCC genotype and phenotype [28]. Thus, UV-related MCC usually exhibits a high TMB in contrast to MCPyV-associated MCC with a low TMB [29].

Excluding cancers with mutations in MMR or polymerase genes, the highest TMB is observed in malignant melanoma, squamous cell carcinomas of the skin, and NSCLC [30, 31]. In this regard, there have been substantial efforts to harmonize and standardize TMB assessment and reporting [23, 24]. The TMB Harmonization Consortium has recently gathered the key stakeholders involved in developing NGS assays reporting their initial results in harmonization and standardization of TMB assessment in cancer [23]. Hopefully, these results will contribute to the standardized approach in assessing TMB across cancers.

### Microsatellite instability (MSI)

DNA MMR machinery in healthy cells is responsible for correcting some errors during DNA replication. The defects in MMR can lead to MSI-H status, which has been demonstrated in at least 14 different cancers at a varying frequency (overall prevalence 2%–4%), most notably in colorectal, gastric, and endometrial cancers [19, 32]. MSI-H or dMMR cancers are characterized by the accumulation of errors in genetic sequences that are usually repeated (these are called microsatellites). Defects in MMR genes (MLH1, MSH2, MSH6, and PMS2) can be hereditary (Hereditary non-polyposis CRC or Lynch syndrome, OMIM#120435) or sporadic (typically caused by hypermethylation of MLH1 gene promoter region) [33]. The dMMR cancers are usually “immunogenic,” exhibiting higher levels of immune cell reaction with higher TIL density than MMR proficient cancers [34]. Consequently, dMMR cancers are more sensitive to ICIs [19].

Based on the study of Le et al. [35] that revealed the predictive value of MSI-H to ICI pembrolizumab irrespective of tumor histology (12 different tumor types were assessed), the MSI-H has been recognized and approved by FDA in 2017 as the first tumor type-agnostic biomarker in the cancer immunotherapy [36]. In addition, both sporadic and hereditary MSI-H CRC tends to express PD-L1 more frequently than MSS CRC [37, 38].

## PD-L1 expression as a predictive biomarker

Multiple studies across the cancers have provided solid evidence about a positive correlation between PD-L1 expression by IHC and response to ICIs [39, 40]. Taube et al. [41] demonstrated that PD-L1 expression on TC was a predictive biomarker for anti-PD-1 drug Nivolumab in 41 patients with advanced solid tumors, including 16 melanoma, 12 NSCLC, 6 CRC, 5 RCC, and 2 patients with castration-resistant prostate carcinoma. In their study, PD-L1 positivity was defined as ≥5% positive TC with membranous PD-L1 expression using two anti-PD-L1 antibodies (5H1 and M3 clones). The study revealed that PD-L1 expression by cancer cells correlated well with an objective response with clinical benefit, while TIL PD-L1 expression was not associated with objective clinical response [41].

In another study, Carbognin et al. [42] explored the correlation between PD-L1 expression and tumor response to three different ICIs, including pembrolizumab, nivolumab, and atezolizumab. The study explored ~1500 patients from 20 clinical trials [42]. They also found that PD-L1 expression in cancer cells in melanoma and NSCLC patients was associated with a higher therapeutic response to ICIs. The positive impact of PD-L1 expression was evident regardless of the treatment approach. In addition, they also showed that the cutoff value of 5% of TC with PD-L1 expression was a better predictor of response to ICIs than the cutoff of 1% of positive cells [42].

In some cancers, such as SCLC, PD-L1, expression was not predictive of response to ICIs, as reported in multiple clinical trials [43, 44]. Consequently, ICIs atezolizumab, pembrolizumab, and durvalumab have been approved in a biomarker-agnostic fashion for patients with this cancer [1]. A poor predictive value of PD-L1 expression by IHC has also been reported in malignant melanoma, hepatocellular carcinoma, and renal cell carcinoma (reviewed in [5]).

### Currently available anti-PD-L1 diagnostic antibodies

We summarized in Tables 1–3 currently available, approved, and commercially available anti-PD-L1 diagnostic antibodies.

**Table 1:** The list of cleared and approved companion diagnostic PD-L1 tests by cancer type (Source: Food and Drug Administration, [1])

**Tumor type**	**Antibody clone (Manufacturer)**	**Scoring algorithms**	**FDA-approved drugs**
Non-small cell lung carcinoma (NSCLC)	VENTANA SP142 Ventana Medical Systems, Inc.*	TC and IC Score	TECENTRIQ (atezolizumab)
	28-8 pharmDx Dako North America, Inc.	TC expression (%)	OPDIVO (nivolumab) combined with YERVOY (ipilimumab)
	22C3 pharmDx Dako North America, Inc.	Tumor Proportion Score (TPS)	KEYTRUDA (pembrolizumab) Libtayo (cemiplimab-rwlc)
Gastric/gastroesophageal junction carcinoma (GEJ)	22C3 pharmDx Dako North America, Inc.	Combined positive score (CPS)	KEYTRUDA (pembrolizumab) Libtayo (cemiplimab-rwlc)
Cervical carcinoma	22C3 pharmDx Dako North America, Inc.	Combined positive score (CPS)	KEYTRUDA (pembrolizumab) Libtayo (cemiplimab-rwlc)
Urothelial carcinoma (bladder)	22C3 pharmDx Dako North America, Inc.	Combined positive score (CPS)	KEYTRUDA (pembrolizumab) Libtayo (cemiplimab-rwlc)
	VENTANA SP142 Ventana Medical Systems, Inc.*	TC and IC score	TECENTRIQ (atezolizumab)
Head and neck squamous cell carcinoma (HNSCC)	22C3 pharmDx Dako North America, Inc.	Combined positive score (CPS)	KEYTRUDA (pembrolizumab) Libtayo (cemiplimab-rwlc)
Esophageal squamous cell carcinoma (ESCC)	22C3 pharmDx Dako North America, Inc.	Combined positive score (CPS)	KEYTRUDA (pembrolizumab) Libtayo (cemiplimab-rwlc)
Triple-negative breast carcinoma (TNBC)	22C3 pharmDx Dako North America, Inc.	Combined positive score (CPS)	KEYTRUDA (pembrolizumab) Libtayo (cemiplimab-rwlc)
	VENTANA SP142 Ventana Medical Systems, Inc.*	IC score	TECENTRIQ (atezolizumab)**

TNBC: Triple-negative breast carcinoma; NSCLC: Non-small cell lung carcinoma; GEJ: Gastroesophageal junction adenocarcinoma; HNSCC: Head and neck squamous cell carcinoma; ESCC: Esophageal squamous cell carcinoma; FDA: Food and Drug Administration; IC: Immune cells; TC: Tumor cells; PD-L1: Programmed cell death ligand 1. * Now Roche Tissue Diagnostics (Tucson, AZ, USA). **Withdrawn voluntarily by Genentech in August 2021.

**Table 2:** Summary of the associated scoring algorithms’ cutoffs and detection platforms for the approved companion diagnostic PD-L1 tests

**Antibody (clone)**	**Scoring algorithms’ cutoff (tumor type)**	**Detection system/platform**
Ventana PD-L1 SP142 Assay	≥5% IC (UC) ≥1% IC (TNBC) ≥50% TC or ≥10% IC (NSCLC)	OptiView Detection and Amplification Benchmark ULTRA
Dako PD-L1 IHC 28-8 pharmDx Assay	≥1% TC (NSCLC)	EnVision Flex-Autostainer Link 48
Dako PD-L1 IHC 22C3 pharmDx Assay	TPS ≥ 1% (NSCLC) CPS ≥ 10 (UC) CPS ≥ 1 (Gastric/GEJ carcinoma) CPS ≥ 1 (cervical carcinoma) CPS ≥ 10 (ESCC) CPS ≥ 1 (HNSCC) CPS ≥ 10 (TNBC)	EnVision Flex-Autostainer Link 48

UC: Urothelial carcinoma; TNBC: Triple-negative breast carcinoma; NSCLC: Non-small cell lung carcinoma; ESCC: Esophageal squamous cell carcinoma; HNSCC: Head and neck squamous cell carcinoma; GEJ: Gastroesophageal junction carcinoma; PD-L1: Programmed cell death ligand 1; IC: Immune cells; TC: Tumor cells.

**Table 3:** Overview of the complementary and other available diagnostic PD-L1 tests

**Antibody (clone/manufacturer)**	**Scoring algorithms’ cutoff (tumor type/drug)**
Ventana PD-L1 SP263 Assay*	≥25% of tumor cells exhibit membrane staining; or, ICP > 1% and IC+ ≥ 25%; or, ICP = 1% and IC+ = 100% (UC) (Durvalumab)
73-10 (Dako Agilent)	Not established yet
E3L1N (Cell Signaling)	Not established yet

PD-L1: Programmed cell death ligand 1. *Complementary assay (FDA)

Although different antibodies against PD-1/PD-L1 are currently available, very few have been approved by the FDA as either companion diagnostic (CDx) or complementary assays (Tables 1–3). A CDx assay is defined as “an *in vitro* diagnostic device (IVD) or an imaging tool that provides information that is essential for the safe and effective use of a corresponding therapeutic product” (List of Cleared or Approved Companion Diagnostic Devices (*In Vitro* and Imaging Tools): Food and Drug Administration; available from [1]). In contrast, a complementary diagnostics test provides additional information about how a drug might be used, which is distinct from CDx tests, which are essential for a drug’s safe and effective use. Most of the available PD-L1 assays have been developed as predictive biomarkers for particular ICIs, each exploring distinct IHC platforms, PD-L1 staining patterns, and scoring systems (algorithms) (Tables 1, 2, and 4) [45, 46].

**Table 4:** Definitions of the currently used scoring systems (algorithms) for the immunohistochemical assessment of PD-L1 expression in cancer

**Scoring algorithm**	**Interpretation**
Tumor Proportion Score (TPS)	The percentage of viable tumor cells showing partial or complete membrane staining relative to all viable tumor cells present in the sample (positive and negative).
Combined Positive Score (CPS)	Number of PD-L1-positive cells (Tumor cells, lymphocytes, and macrophages) divided by the total number of viable tumor cells in the assessed area, multiplied by 100.
Tumor cells (TC) Score	The percentage of PD-L1-positive tumor cells at any intensity.
Immune cells (IC) Score	The proportion of tumor area occupied by PD-L1-positive immune (mononuclear) cells at any intensity.

PD-L1: Programmed cell death ligand 1.

PD-L1 expression can be seen in cancer and IC infiltrating invasive cancer (both in intra- and peritumoral stroma) [37, 45]. However, the assessment of IC includes only mononuclear infiltrate (lymphocytes, macrophages, and dendritic cells), while plasma cells and neutrophils should be ignored.

**Figure 1.**
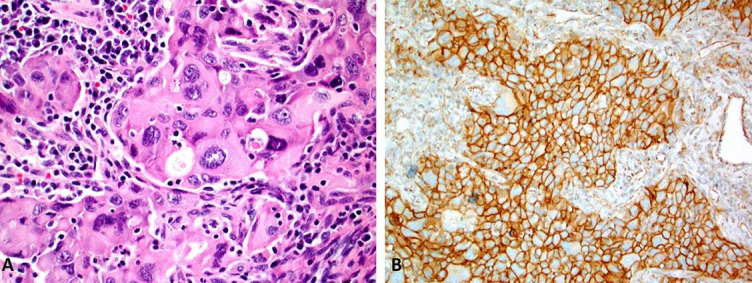
**A morphology (Image A; magnification 40×) of poorly differentiated pulmonary non-small cell lung cancer with a marked nuclear atypia with diffuse and strong membranous PD-L1 expression (Image B; SP142 clone, magnification 20×).** PD-L1: Programmed cell death ligand 1.

**Figure 2.**
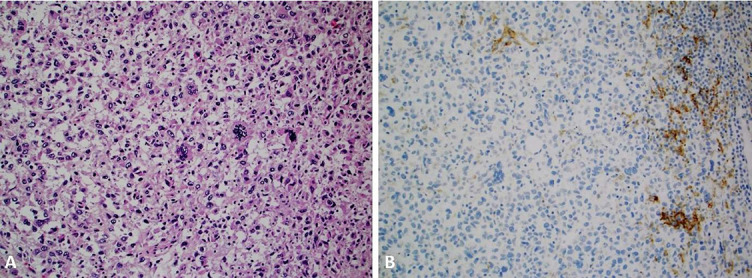
**A high-grade triple-negative breast cancer case with metaplastic and pleomorphic features (Image A, magnification 20×) with PD-L1 expression in the tumor-infiltrating lymphocytes (SP142 clone) [99, 100].** The tumor cells were devoid of PD-L1 expression (Image B, magnification 20×). PD-L1: Programmed cell death ligand 1.

**Figure 3.**
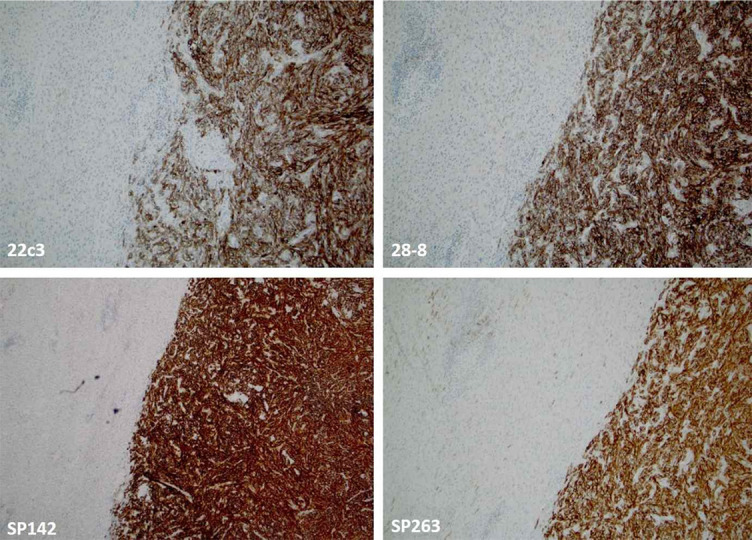
**There is an excellent concordance between the four clones in assessing PD-L1 expression in metastatic soft tissue neoplasm (dedifferentiated liposarcoma) (magnification 10×).** PD-L1: Programmed cell death ligand 1.

**Figure 4.**
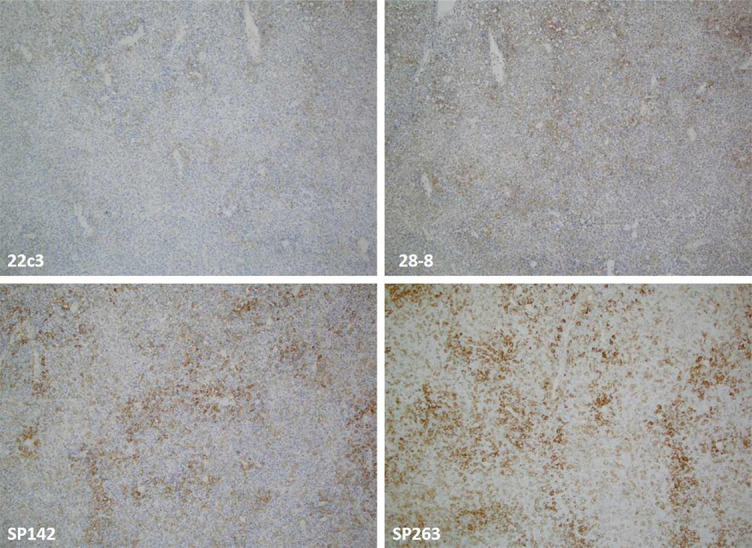
**A case of diffuse large B-cell lymphoma (DLBCL) exhibiting a discordant expression (in both intensity and percentage) of PD-L1 with the lowest expression with the 22C3 and the highest with SP263 clone (magnification 10×).** PD-L1: Programmed cell death ligand 1.

The staining pattern of PD-L1 differs between cancer and IC. The neoplastic cells typically exhibit a linear membranous PD-L1 pattern (Figure 1), while the IC have granular and punctate PD-L1 expression (Figure 2). This distinction is particularly relevant in cancers when assessing exclusively IC for PD-L1 (e.g., triple-negative breast cancer). Although all the available clones exhibit similar subcellular patterns of PD-L1 expression in cancer and IC, respectively (Figures 3 and 4), significant differences in measurements exist [47].

The following paragraphs summarize the most relevant information on the approved CDx for PD-L1 testing.

### Clones available from Roche Tissue Diagnostics, Tucson, AZ (formerly, Ventana Medical Systems, Inc.): SP142 and SP263 assays

Both assays are monoclonal rabbit antibodies recommended and optimized for use on the Ventana BenchMark Ultra instrument. SP142 has been approved as a CDx for multiple malignancies (NSCLC, urothelial carcinoma, and triple-negative breast carcinoma); however, each of the provided indications has different cutoffs and scoring (interpretation) systems (Tables 1, 2, and 4). In contrast, SP263 has only been approved as a complementary assay for predicting the urothelial (bladder) carcinoma response to durvalumab (ICI against PD-L1) [48] (Table 3).

### Clones available from Agilent DAKO Products: 22C3 pharmDx assay, 28-8 pharmDx assay, and 73-10 assay

Both 22C3 pharmDx and 28-8 pharmDx are FDA-approved diagnostic PD-L1 assays for multiple malignancies (Tables 1 and 2). In particular, the 22C3 clone has been utilized as a predictive biomarker for several cancers, including NSCLC, urothelial (bladder) carcinoma, cervical carcinoma, gastroesophageal/gastric (GEJ) carcinoma, esophageal squamous cell carcinoma (ESCC), triple-negative breast carcinoma, and head and neck squamous cell carcinoma (HNSCC) (Tables 1 and 2). Similar to the SP142 assay, the 22C3 clone also has different cutoff and cancer-specific scoring algorithms (Tables 3 and 4). The 28-8 pharmDx assay has been primarily utilized for NSCLC (Tables 1 and 2).

The third clone is 73-10. It has initially been developed for clinical trials exploring the anti-PD-L1 agent avelumab. Despite good analytical performance and concordance with other anti-PD-L1 antibodies and confirmed predictive value in NSCLC [49–51], the 73-10 clone has not yet been approved by FDA (Table 3). The threshold for positivity and scoring algorithm is also to be determined for this antibody.

### Scoring algorithms

Four different scoring systems (algorithms) have been proposed and validated for the PD-L1 assessment and quantification by IHC so far. These include the TC score, IC score, tumor proportion score (TPS), and combined positive score (CPS) (their definitions are summarized in Table 4). Each scoring algorithm has been designed and approved for the specific ICIs (Table 1).

TPS (%) is defined as the percentage of viable cancer cells with partial or complete membrane expression (≥1+) relative to all viable cancer cells present in the entire sample (positive and negative).

CPS is calculated as the number of PD-L1 positive cells (both cancer and IC) divided by the total number of viable cancer cells multiplied by 100.

TC score (%) implies the number of PD-L1-positive cancer cells divided by the total number of cancer cells.

IC score (%) is expressed as the total number of PD-L1-positive mononuclear cells (lymphocytes and macrophages) at any intensity within the tumor area. The tumor area includes intra- and peritumoral stroma. The expression of PD-L1 in cancer cells is not considered for the IC score.

Multiple studies have evaluated the concordance (inter-assay heterogeneity) between the different PD-L1 clones in common cancers revealing highly variable results [52–63]. For instance, in the IMpassion130 trial, 46% of TNBC samples were positive with SP142 clone (Ventana); when another assay (22C3, Agilent) was utilized on the same samples, the positivity significantly increased (~80%) [64]. However, a recent comprehensive systematic review of Prince et al. [65] revealed an excellent concordance between 28-8, 22C3, and SP263 clones in assessing PD-L1 expression in cancer cells among the common cancer subtypes (e.g., NSCLC, HNSCC, and urothelial carcinoma); in contrast, SP142 clone stained substantially fewer cancer cells than the three other clones in these cancers. Notably, the concordance between the four clones was substantially lower for PD-L1 assessment in IC [65].

Similar findings were reported in another systematic review with a meta-analysis conducted by Torlakovic et al. [66]. The study explored the diagnostic accuracy of the laboratory-developed PD-L1 assays. It also revealed that the Ventana SP142 assay’s analytical sensitivity was significantly lower than the three other FDA-approved PD-L1 assays in NSCLC and some other cancers (22C3 pharmDx, 28-8 pharmDx, and Ventana SP263 assays). The authors of the meta-analysis concluded that “fit-for-purpose” PD-L1 laboratory-developed assays (particularly in referral and expert-led laboratories) might be comparable with the PD-L1 FDA-approved assays (CDx and complementary assays) when both types of assays are compared with an appropriately designated reference standard [66]. In line with these findings are recent recommendations from the Canadian Association of Pathologists-Association Canadienne Des Pathologistes (CAP-ACP) regarding the fit-for-purpose PD-L1 assay development and optimization for selecting the patients in I-O [67]. Figures 3 and 4 illustrate the performance of four (SP142, SP263, 22C3, 28-8) anti-PD-L1 assays [68].

### Practical considerations, potential pitfalls and refinements in immunohistochemistry assessments of immune checkpoint blockade

As shown above, several cancers are currently routinely tested for PD-L1 expression using IHC [69]. However, this method is not absolute regarding the predictive utility of PD-L1 expression of responsiveness to ICIs, particularly monotherapy [69, 70]. It is also well known that some patients whose cancers exhibit PD-L1 expression may not have a therapeutic response to ICIs, while some patients with negative PD-L1 test may still be responsive to ICIs [39]. These facts represent potential caveats predicting therapeutic response through PD-L1 assessment [70–72].

Several important limitations associated with PD-L1 IHC testing should be mentioned: a) Sensitivity of the commercially available assays (CDx), as demonstrated in several studies; b) Various issues related to IHC assay and methodology; and c) Tissue sampling and preparation. Recently published recommendations of pharmaceutical and *in vitro* CDx industries on one side and the Personalized Health Care Committee of the College of American Pathologists (CAP) on the other recognized an unmet need to harmonize the design and utility of various CDx in a clinical setting [46]. Other researchers also recognized this problem, highlighting the need for PD-L1 IHC standardization and harmonization in clinical practice [73–75]. Recent technology advances (digital pathology and deep learning/artificial intelligence/AI/) with computer-assisted PD-L1 assessment and scoring may be good solutions to overcome the shortcomings related to manual PD-L1 evaluation [76, 77]. A recent, multi-institutional study explored the utility of the AI-assisted method in the evaluation of PD-L1 IC expression in breast cancer (SP142 clone) [78]. The proposed AI tool substantially improved the accuracy and concordance in PD-L1 interpretation, contributing to a better PD-L1 standardization in clinical practice [78]. Several studies analyzing the AI assistance in PD-L1 assessment in NSCLC [79–83] and HNSCC [84] have been recently published.

In addition, intratumoral PD-L1 expression is very complex and dynamic. PD-L1 status may be genetically upregulated in cancer cells, e.g., via PD-L1 (CD274) gene amplification (a good example is Hodgkin lymphoma) [68]. In addition, PD-L1 expression is tightly regulated at different molecular levels, including transcriptional, posttranscriptional, and protein levels [85]. Furthermore, the presence of PD-L1 positive TC or IC may differ in different parts of the tumor as well as it may differ between primary and metastatic sites (e.g., NSCLC with different microenvironment in the primary and metastatic sites /brain/) [86, 87]. Consequently, single slide PD-L1 IHC may be an insufficient method to fully reflect the dynamic PD-L1 expression in cancer [71]. Davis and Patel [88] assessed the predictive utility of PD-L1 expression based on all ICIs approved by the FDA through 2019. They found a low predictive value (~29%) of PD-L1 positivity, while in the remaining cases, PD-L1 expression was either not predictive (53%) or not tested (18%) [88].

Broad and comprehensive reviews on this important topic have already been published by Nimmagadda [89] and Cottrell and Taube [8]. A recent study on the predictive value of PD-1 IHC (coupled with image analysis) in patients undergoing treatment with PD-1 inhibitors (nivolumab and pembrolizumab) for NSCLC patients showed that PD-1 density is a better predictive biomarker for durable clinical benefit in these two NSCLC cohorts treated with PD-1 blockade than PD-L1 score [90]. It is important to note that presence of PD-1-positive TILs was observed in many cancer types beyond NSCLC and was generally associated with the increased number of mutations in TC [37].

From the practical point of view, it is essential to highlight that PD-L1 expression in TC is considered positive regardless of the completeness of the membranous staining; however, in gland-forming cancers (e.g., adenocarcinomas), staining confined exclusively to the luminal border is considered negative. On the other hand, both membranous and cytoplasmic expressions in IC are considered positive. Intratumoral macrophages may also overexpress PD-L1, including macrophages within glandular lumens; however, this staining should not be counted as PD-L1 positivity. Similar to other IHC stains, intracellular pigments in some cancers (e.g., melanin, hemosiderin, and anthracotic pigment) can occasionally make the interpretation of PD-L1 staining difficult [91].

Other essential aspects relevant when considering the predictive value of PD-L1 testing include inter- and intra-tumoral heterogeneity with variable effects on PD-L1 expression [54]. Sample details (primary vs. metastatic cancer, sample age, sample type/small/core biopsy vs. large/surgical/ biopsy, naïve vs. treated samples) and various preanalytical variables [e.g., time of collection relative to treatment testing/”age” of the specimen, time to fixation, type of fixative, fixation time (cold ischemia time), decalcification for bone specimens] may have a significant impact on PD-L1 expression and interpretation [5]. In particular, bone samples may not be suitable for PD-L1 assessment as currently approved CDx, such as SP142 and SP263, are only validated for non-decalcified samples fixed in 10% neutral-buffered formalin. Similarly, CDx for PD-L1 is not validated on cytology samples, including cellblocks, despite recent studies that confirmed an excellent concordance between biopsy and cytology samples in some cancers such as NSCLC [92, 93].

Mansour et al. [94] also demonstrated an excellent PD-L1 concordance between cytology and histology (biopsy) samples in NSCLC. Their comprehensive literature survey, based on 25 published studies with ~1700 paired cytopathology/histopathology samples, showed the median (range) concordance between 81% and 85% (62%–100%) at a threshold of 1% for a positive PD-L1 staining and 89% (67%–100%) at the threshold of 50% [94]. However, they also found significant variations between laboratories, stressing the importance of optimization and quality assurance in PD-L1 testing [94].

Like all other IHC stains, a lab that performs PD-L1 staining must be continuously involved in external quality assurance of the assay (e.g., NordiQC, UK NEQAS, and CAP) [95].

Several studies also explored the effects of chemotherapy on PD-L1 expression in cancers [96–98], reporting the upregulated effects of cytotoxic therapy on PD-L1 expression. In lung cancer, this upregulation of PD-L1 was associated with an adverse clinical outcome [96], while in some other cancers (e.g., ESCC), PD-L1 activation following chemotherapy had no impact on patients’ outcomes [98].

## Conclusion

Immunotherapy based on immune checkpoint inhibitors has substantially improved the outcome and prognosis of numerous common malignancies. Several predictive biomarkers have been validated and approved, including PD-L1 testing by IHC as the most widely used predictive biomarker. Despite its evident clinical utility and CDx status by FDA for several cancers, the predictive value of PD-L1 expression remains low across cancers. In addition, PD-L1 testing and interpretation are not easy to perform due to the unique IHC assays and interpretations for each immune checkpoint inhibitor and various preanalytical issues common for all IHC assays. Additional efforts from industry, stakeholders/regulatory bodies/, academia, and clinicians are necessary to harmonize and improve the overall PD-L1 IHC performance and provide novel predictive biomarkers to immune checkpoint inhibitors.

**Conflicts of interest:** The authors declare no conflicts of interest.

**Funding:** The authors received no specific funding for this work.
